# Deciphering the Role of microRNAs in Large-Artery Stiffness Associated With Aging: Focus on miR-181b

**DOI:** 10.3389/fphys.2021.747789

**Published:** 2021-09-27

**Authors:** Jay M. Baraban, Eric Tuday, Dan E. Berkowitz, Sam Das

**Affiliations:** ^1^Department of Neuroscience, School of Medicine, Johns Hopkins University, Baltimore, ML, United States; ^2^Division of Cardiovascular Medicine, Department of Internal Medicine, School of Medicine, University of Utah, Salt Lake City, UT, United States; ^3^Geriatric Research, Education and Clinical Center, VA Salt Lake City Health Care System, Salt Lake City, UT, United States; ^4^Department of Anesthesiology and Perioperative Medicine, School of Medicine, University of Alabama at Birmingham, Birmingham, AL, United States; ^5^Department of Pathology, School of Medicine, Johns Hopkins University, Baltimore, ML, United States; ^6^Department of Anesthesiology and Critical Care Medicine, Johns Hopkins Medicine, Baltimore, ML, United States

**Keywords:** miR-181b, translin/trax, microRNA degradation, arterial stiffness, aging

## Abstract

Large artery stiffness (LAS) is a major, independent risk factor underlying cardiovascular disease that increases with aging. The emergence of microRNA signaling as a key regulator of vascular structure and function has stimulated interest in assessing its role in the pathophysiology of LAS. Identification of several microRNAs that display age-associated changes in expression in aorta has focused attention on defining their molecular targets and deciphering their role in age-associated arterial stiffening. Inactivation of the microRNA-degrading enzyme, translin/trax, which reverses the age-dependent decline in miR-181b, confers protection from aging-associated arterial stiffening, suggesting that inhibitors targeting this enzyme may have translational potential. As LAS poses a major public health challenge, we anticipate that future studies based on these advances will yield innovative strategies to combat aging-associated arterial stiffening.

## Introduction

The emergence of Large-Artery Stiffness (LAS) as a major risk factor for cardiovascular morbidity has stimulated interest in developing strategies to combat this endemic public health challenge (O'Rourke, 1990; Mitchell et al., 2010; Boutouyrie et al., 2011; O'Rourke et al., 2016; Chirinos et al., 2019). Under normal conditions, the elasticity of the aorta and other major arteries absorbs and dampens the pressure pulsation generated during systole. However, the increased stiffness of these vessels in patients with LAS disrupts the normal hemodynamics of the cardiac cycle, allowing conduction of waves with abnormally high pressure into the vascular beds of target organs, such as kidney and brain, that contribute to tissue damage.

## Identification of Candidate microRNAs Implicated in LAS

Arterial stiffness is determined by the composition and organization of the extracellular matrix, as well as the cytoskeletal properties of vascular smooth muscle cells (VSMCs; Qiu et al., 2010; Sehgel et al., 2015; Chirinos et al., 2019). Therefore, investigators have concentrated on defining the biochemical and associated structural changes responsible for increased arterial stiffness, as well as the upstream regulatory pathways driving these changes. From this perspective, the microRNA system has emerged as a potential culprit, since this highly versatile signaling pathway can coordinate cellular adaptations in response to developmental and environmental signals (Bartel, 2004) and has been found to play key roles in vascular smooth muscle development and function (Albinsson et al., 2010; Kang and Hata, 2012). However, since each cell contains hundreds of distinct microRNAs, and these microRNA profiles differ across cell types, identifying candidate microRNAs involved in regulating arterial stiffness poses a difficult challenge.

Initial clues implicating dysregulation of specific microRNAs in LAS emerged from two approaches. As patients with LAS can be identified by measuring pulse wave velocity (PWV) using non-invasive techniques, investigators looked for alterations in microRNA signaling associated with elevated PWV. This approach yielded identification of two microRNAs linked to elevated PWV: miR-765 and (Liao et al., 2015), miR-1185 (Deng et al., 2017). The second approach was based on the epidemiological observation that the prevalence of LAS increases markedly with aging (O'Rourke and Nichols, 2005; Mitchell et al., 2007; Chirinos et al., 2019). Furthermore, mice also display aging-associated increases in PWV that model LAS (Nicholson et al., 2017; Steppan et al., 2019). Thus, investigators checked for microRNAs that show altered expression with aging in blood samples from humans and in mouse aorta. This approach led to the identification of several candidate microRNAs that show altered expression with aging: mir-29, miR-34a, miR-92a, miR-137, miR-181b, miR-203, and miR-222. Mir-29, miR-34a, miR-137, miR-203, and miR-222 increase with aging in mouse aorta (Boon et al., 2011; Badi et al., 2015; Nicholson et al., 2017) and miR-34a has been shown, very recently, to be associated, along with miR-34c, with aortic stiffening in human subjects (Gatsiou et al., 2021). Conversely, miR-92a and miR-181b decrease in mouse aorta with aging (Hazra et al., 2016; Hori et al., 2017) and miR-92a is also decreased in human blood with aging (Hazra et al., 2016).

Based on these initial observations, investigators examined whether mimicking these changes is sufficient to produce stiffening. Using a transfection-based approach *in vivo*, Nicholson et al. (2017) found that elevating miR-203 leads to arterial stiffening. Furthermore, administering an antagonist of miR-92a to mice increases PWV (Hazra et al., 2016). To examine the effect of decreased miR-181b on aortic stiffness, Hori et al. (2017) used mice carrying a deletion of the locus (miR-181a1/b1) that blocks expression of both miR-181a and miR-181b in aorta. These mice showed a premature onset of arterial stiffness as young adults. Thus, these findings indicate that aging-associated changes in expression of these candidate microRNAs play a causal role in eliciting arterial stiffness.

## Organization of the microRNA Signaling System Confers Versatility

Prior to discussing how altered expression of these microRNAs might contribute to LAS, we will first provide a brief overview of the operating principles of the microRNA signaling pathway (Bartel, 2004; Friedman et al., 2009; McGeary et al., 2019). MicroRNAs are ~20–22 nucleotide fragments that are generated from larger RNA transcripts that undergo two processing steps. The primary transcript is cleaved into one or more hairpin-shaped fragments in the nucleus. These hairpin-shaped fragments, called pre-microRNAs, are then exported from the nucleus into the cytoplasm, where they are cleaved by Dicer, an RNA endonuclease, into mature microRNAs, double-stranded oligomers with 3' overhangs. One of these strands, called the guide strand, binds to Ago, a protein component of the RNA-induced silencing complex (RISC). The guide strand targets the RISC complex to mRNAs that contains its complementary sequence in its 3'UTR, where it acts to prevent its translation or trigger its degradation. By using guide microRNAs to target specific mRNAs, this system has tremendous versatility. Each species of microRNA can target multiple mRNAs to regulate their translation; conversely, each mRNA can be regulated by multiple microRNAs.

While the architecture of the microRNA signaling pathway appears to be designed to maximize versatility, it is also important that it have the capacity for selectivity, the ability of the cell to enhance or dampen the impact of individual or a small cohort of microRNAs. Since Dicer plays an essential role in generating virtually all microRNAs, regulating its activity would not provide a mechanism to generate selectivity as it would produce a global change in microRNA production. Instead, two alternative mechanisms are used to achieve selectivity. One depends on selective transcription of the primary transcripts. Like mRNAs, microRNAs are also transcribed by Pol II. Thus, their expression is regulated by the same cohort of promoters used to regulate coding transcripts. This promoter system enables each cell type to express a different profile of microRNAs and alter their levels in response to changes in cellular conditions. The other depends on selective degradation of microRNAs, which is achieved by two distinct mechanisms: one involves conventional RNase enzymes (Fu et al., 2016; Baraban et al., 2018) while the other involves a novel RNA-guided mechanism.

## microRNA Degradation Pathways Confer Selectivity

The best characterized microRNA-degrading pathway is mediated by Lin28, which targets the let-7 group of pre-microRNAs (Heo et al., 2008, 2009; Hagan et al., 2009). These pre-microRNAs contain a common motif in their loop that is recognized by Lin28. Binding of Lin28 to let-7 pre-miRNAs triggers addition of uridine residues to its 3'terminal, which targets these pre-miRNAs for degradation.

The translin (TN)/trax (TX) microRNA-degrading enzyme is another RNase that targets a subset of pre-microRNAs (Asada et al., 2014). The target specificity of the TN/TX RNase has not been as well characterized as that of Lin28. However, limited characterization of its substrate specificity indicates that it acts at mismatches in base pairing located in the stem, as mutations that eliminate selected mismatches abolish cleavage. Comparison of microRNA profiles from wild type tissues or cells with those that are devoid of TN/TX activity has yielded identification of a small subset of microRNAs that are elevated by this manipulation and hence considered candidate substrates.

A third microRNA-degrading enzyme, called monocyte chemotactic protein-induced protein (MCPIP), has been identified in studies on immune cells (Suzuki et al., 2011). Initial characterization of its substrate specificity has demonstrated that it, like Lin28, recognizes a motif in the loop of pre-microRNAs. Further studies are needed to define its role in regulating microRNA signaling.

One of the potential advantages of using RNases with a high degree of selectivity to regulate microRNA levels is that they can cause rapid decreases in the levels of a small subset of target microRNAs. Elegant studies from the Meffert lab have demonstrated that rapid activation of the Lin28 degradation pathway is used by BDNF and other growth factors to trigger rapid increases in translation of mRNAs that mediate cellular plasticity changes mediated by these growth factors (Huang et al., 2012; Amen et al., 2017). At the same time, these growth factors also increase Dicer activity to produce a global increase in generation of microRNAs and subsequent translational silencing of mRNAs that are not targeted by the Lin28/let-7 pathway. In a similar vein, several forms of synaptic plasticity in hippocampal slices are dependent on TN/TX-mediated degradation of a few microRNAs, which triggers *de novo* translation of plasticity transcripts (Park et al., 2017, 2020). Thus, activation of these microRNA-degrading enzymes provides a mechanism to trigger selective increases in protein translation by reversing microRNA-mediated silencing.

Based on these findings with Lin28, TN/TX, and MCPIP, we and others have speculated that there may be many other microRNA-degrading enzymes that remain to be discovered that target discrete subsets of microRNAs. However, this may not be the case because recent studies have uncovered a novel RNA-based mechanism for selective degradation of microRNAs (Sheu-Gruttadauria et al., 2019). This process was initially discovered as a means by which a viral encoded mRNA triggers degradation of a specific host cell microRNA (Cazalla et al., 2010). In this process, called target-mediated degradation of microRNAs (TMDM), the viral transcript binds with a high degree of complementarity to the target microRNA. At first glance, it is unclear how this initial step in the degradation process differs from what occurs in the silencing process. However, a more detailed analysis of the degree of complementarity between the guide microRNA and its target sequence has revealed a critical difference. In the silencing process, a microRNA guides Ago to a target mRNA by a region of complementarity in the 5' portion of the microRNA. However, in TMDM, the mRNA transcript co-opts this process by binding to the guide microRNA at both its 5' and 3' ends. This fairly subtle difference in the degree of complementarity between microRNA and its target mRNA has a huge impact on the outcome of this interaction. Rather than the microRNA silencing the mRNA, the mRNA triggers degradation of the microRNA. Thus, by using RNA-mediated recognition of microRNAs, cells can produce degradation of selected microRNAs without the need for additional RNase enzymes (Han et al., 2020; Shi et al., 2020).

## Molecular Targets of Candidate microRNAs

As microRNAs act by suppressing translation of target mRNAs, identification of microRNAs implicated in LAS has stimulated interest in identifying their direct mRNA targets (Table 1). Using bioinformatic approaches, candidate target mRNAs for these microRNAs were identified by the presence of complementary binding sites in their 3'UTR. Using this search strategy, elastin, fibrillin, and collagens 1A1 and 3A1 have been identified as targets of miR-29 (van Rooij et al., 2008; Boon et al., 2011). Since miR-29 levels increase with aging, expression of these target proteins decline, as expected. Furthermore, administration of an miR-29 antagonist *in vivo* has the opposite effect. Using a similar approach, sirtuin 1 (SIRT1), which has been linked to preventing cellular senescence, was identified as a target of miR-34a in VSMCs (Badi et al., 2015). According to this scenario, the elevation in miR-34a inhibits SIRT1 expression, thereby enhancing cellular senescence, which contributes to stiffening. Conversely, miR-92a which decreases with aging has been shown to target collagen 1 and TNF-alpha receptor 1 (Hazra et al., 2016). Direct targets identified for miR-181b, which decreases with aging, are elastin and tissue inhibitor of metalloproteinase-3 (TIMP-3) (Di Gregoli et al., 2017), as well as TGF-βi, an uncharacterized gene product that is induced by TGF-β (Hori et al., 2017). Lastly, miR-34a, miR-137, miR-203 and miR-222, which increase with aging, have been shown to target components of the pathway that mediate Src-dependent cytoskeletal remodeling in VSMCs, a process that can reduce agonist-induced VSMC stiffness *in vitro*. Therefore, heightened expression of these microRNAs could contribute to increased VSMC stiffness in aging by impairing this signaling pathway which promotes aortic plasticity (Nicholson et al., 2017).

**Table 1:** MicroRNAs that show altered expression in aorta with aging.

**miRNA**	**Δ with aging**	**Candidate targets**
miR-29	Up	Elastin, fibrillin, collagen 1A1/3A1
miR-34a	Up	SIRT1, Src, FAK, paxillin, vinculin
miR-137	Up	Src, paxillin
miR-203	Up	Src, Crk, paxillin, talin
miR-222	Up	Crk, FAK
miR-92a	Down	Collagen 1, TNFalpha R1
miR-181b	Down	Elastin, TIMP-3, TGF-βi

## Testing the Role of miR-181b in LAS

An important goal of deciphering the pathophysiology of LAS is to identify potential therapeutic strategies that can be used to prevent or reverse this disorder. Accordingly, a critical step in evaluating the role of candidate microRNAs in LAS is testing whether manipulations that normalize levels of a candidate microRNA during aging decrease arterial stiffness in an animal model of LAS. Although several studies have demonstrated that mimicking the alteration of a candidate microRNA is sufficient to elicit arterial stiffness, to our knowledge, miR-181b is the only candidate microRNA that has been subjected to this rigorous test.

Our strategy for normalizing miR-181b levels in aorta emerged from our observation that this microRNA is one of those elevated in a cell line that had been subjected to knockdown of TN/TX, suggesting that it was targeted by this microRNA-degrading enzyme (Asada et al., 2014). To confirm this inference, we first checked whether miR-181b levels are elevated in aorta harvested from TN KO mice and found this to be the case (Tuday et al., 2019). Therefore, we reasoned that if a decrease in miR-181b levels plays a key role in driving LAS, then TN KO mice might be resistant to developing arterial stiffness. As C57BL6 mice display aging-associated arterial stiffening, they provide a suitable animal model of this disorder (Nicholson et al., 2017). However, since that would require waiting until the mice are close to 18 months old, we first tested this hypothesis in a paradigm that elicits increased aortic stiffness in just a few weeks. In this streamlined paradigm, mice are switched from regular water to high salt water (HSW; 4% w/v), and their PWV measured on a weekly basis. Remarkably, we found that TN KO mice are resistant to developing increased PWV induced by this paradigm. Furthermore, exposure to HSW decreases levels of miR-181b in WT mice, but not in TN KO mice, consistent with the view that TN deletion confers protection from increased stiffness by blocking degradation of miR-181b. However, it is important to emphasize that this *in vivo* study leaves open a variety of alternative possibilities. For example, it is possible that the protective effect of TN deletion is due to blocking degradation of other miRNAs during the HSW paradigm.

**Figure 1:**
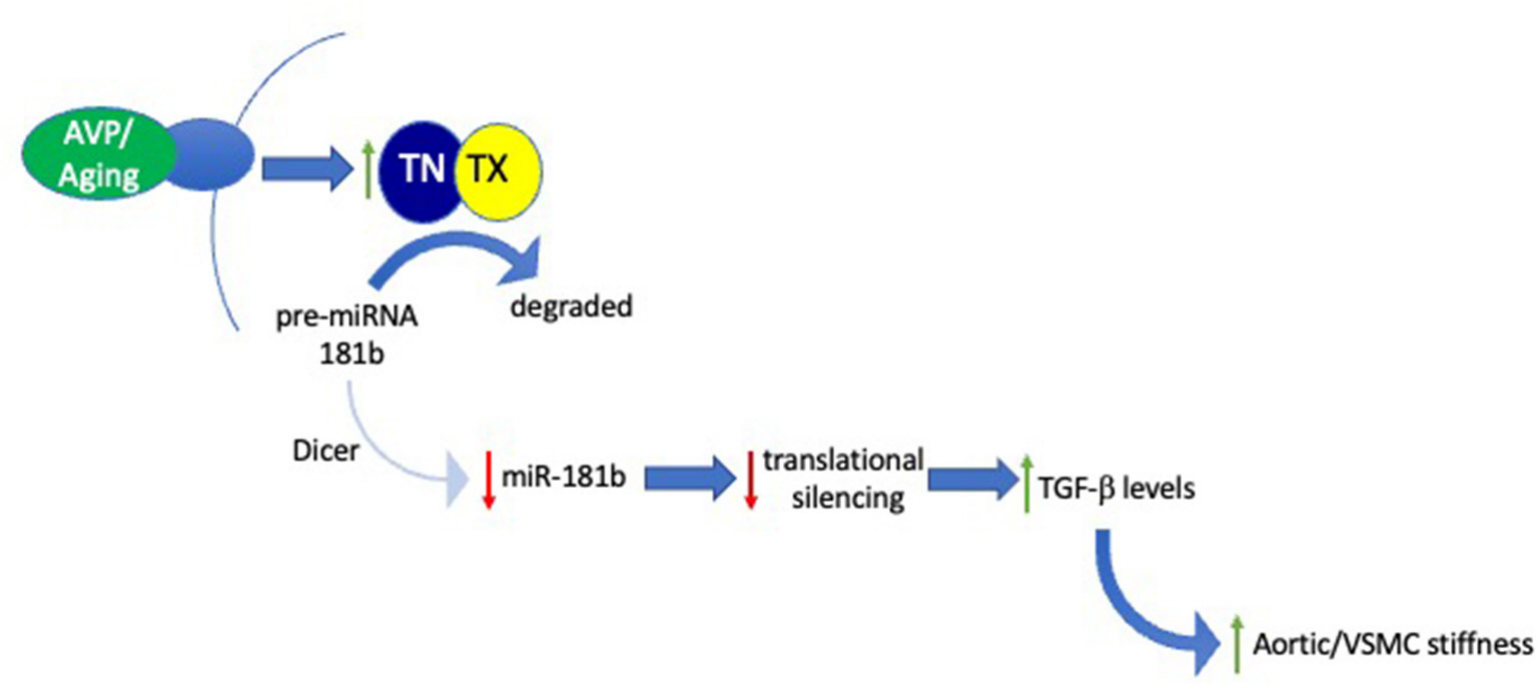
Signaling pathway mediating role of the TN/TX microRNA-degrading enzyme in regulating aortic stiffness. Schematic diagram integrates results obtained in both *in vitro* and *in vivo* studies examining the role of TN/TX and miR-181b in mediating VSMC and aortic stiffness. In this model, aging or AVP stimulation elicit activation of TN/TX which cleaves pre-miR-181b, the precursor of mature miR-181b. Degradation of pre-miR-181b prevents Dicer from generating mature miR-181b. The subsequent decrease in levels of mature miR-181b blocks its ability to silence translation of its mRNA targets. Although the identity of these mRNA targets remains to be identified, we hypothesize that allowing them to be translated by reducing miR-181b levels leads to increased extracellular levels of TGF-β. According to this model, excess TGF-β signaling drives the increase in VSMC and ECM stiffness underlying LAS.

To test our hypothesis that miR-181b plays a key role in regulating arterial stiffness, we have, more recently, examined the impact of manipulating miR-181b in an *in vitro* model of VSMC stiffness (Tuday et al., 2021). In this paradigm, exposure of VSMCs to arginine vasopressin (AVP) for several hours elevates their stiffness, as determined by magnetic twist cytometry, and reduces miR-181b levels in a TN/TX-dependent fashion. Thus, this model provided an excellent opportunity to test whether the increased stiffness produced by AVP is mediated by its ability to reduce miR-181b levels. To check this key point, we found that transfecting cells with exogenous miR-181b blocked the ability of AVP to increase stiffness. Furthermore, we found that the AVP-induced reductions in miR-181b trigger increased VSMC stiffness by increasing levels of TGF-β in the media, as neutralizing antibodies to TGF-β block AVP-induced increases in VSMC stiffness in this paradigm (Figure 1).

To assess whether TN/TX inactivation can also confer protection against arterial stiffness associated with aging, we checked whether mice containing a point mutation in TX, E126A, which abolishes the RNase activity of the TN/TX RNase (Fu et al., 2020), display protection in this paradigm. As expected, we found that that these mutant mice are protected from developing arterial stiffness with aging. As aging-induced arterial stiffness is associated with increased vessel wall thickness, it is also noteworthy that aorta wall thickness in aged TX(E126A) mice is thinner than in aged WT mice. Thus, these studies strongly indicate that deletion or inactivation of the TN/TX microRNA degrading enzyme confers protection against arterial stiffening *in vivo*, and that it does so by blocking the reduction in miR-181b observed with aging.

## Role of miR-181b in Other Vascular Pathology Paradigms

MiR-181b has also been studied in other paradigms of vascular pathology. Whereas the studies outlined above indicate that reduced levels of miR-181b contribute to arterial stiffness and that reversing that decrease has therapeutic effects, Di Gregoli et al. (2017) report that miR-181b levels are elevated in human atherosclerotic plaques and abdominal aortic aneurysms and that *in vivo* administration of miR-181b inhibitors has beneficial effects in mouse models of both these pathologies. These investigators provide compelling evidence that tissue inhibitor of metalloproteinase-3 (TIMP-3) is a direct target of miR-181b in macrophages present in atherosclerotic plaques and in abdominal aortic aneurysms that mediates its deleterious effects. Furthermore, administration of an miR-181b inhibitor *in vivo* to ApoE KO mice, which are prone to developing aneurysms, alters the composition of the extracellular matrix by decreasing the presence of new collagen and increasing the amount of elastin. As bioinformatic analysis indicates that elastin is a direct target of miR-181b, these findings would fit with the predicted effects of miR-181b inhibition.

On the surface, the findings of Di Gregoli et al. (2017) appear to be at odds with those of Tuday et al. (2021). The latter indicates that elevation of miR-181b by inhibiting its degradation via TN/TX protects against age-associated arterial stiffness; the former suggests that miR-181b inhibition protects against development of atherosclerotic plaques and aortic aneurysms. However, one straightforward way of integrating these findings is that normalizing the levels of miR-181b in each of these paradigms leads to a beneficial result. In aging, when miR-181b levels are low, increasing them by inhibiting TN/TX is beneficial. In models of atherosclerotic plaque and aortic aneurysms which display marked elevations of miR-181b, then selective inhibition of this microRNA is protective. Another common feature of these results is that manipulating miR-181b has striking effects on key components of the extracellular matrix, reinforcing the view that strategies aimed at regulating its expression provide an effective means of influencing arterial stiffness.

## Summary and Future Directions

Taken together, the studies summarized above provide compelling evidence that alterations in microRNA signaling in aorta play a key role in the development of LAS. In this review, we have focused on their role in VSMCs. However, there is also a large body of literature documenting their important roles in regulating endothelial cell function (Wei et al., 2013; Cao et al., 2020). Thus, an important next step in this field will be to assess whether manipulating miRNA levels in VSMCs selectively is sufficient to induce or prevent arterial stiffening or whether parallel processes in endothelial cells or other cells present in large vessels, such as immune cells or fibroblasts, also play a key role in the pathophysiology of LAS.

Comparison of the longitudinal development of arterial stiffness with aging in males and females has revealed a prominent increase in post-menopausal women (Coutinho, 2014; DuPont et al., 2019), suggesting that estrogen has a protective effect. The exact mechanism in human pathophysiology is not fully understood; however, animal studies suggest significant effects of estrogen on the extracellular matrix as a primary contributor (Ogola et al., 2018). The growing evidence for a role of microRNA dysregulation in the etiology of this disorder provides a strong rationale for assessing whether these epidemiological findings reflect hormonal effects on microRNA signaling. Furthermore, since aging male mice develop increased PWV (Nicholson et al., 2017), we now emphasize the need for a comprehensive female animal longitudinal study as it would be interesting to check whether female aging mice might provide a useful model to investigate mechanisms underlying increased LAS in post-menopausal women.

As inhibiting the activity of the TN/TX microRNA-degrading enzyme confers protection from the development of aging-associated arterial stiffening, inhibitors of this enzyme may have translational potential. However, since the observed protection occurred in mice with a constitutive inactivation of TN/TX, it will be important to check if inhibition of this enzyme after the initial onset of aging-associated arterial stiffening can prevent the progression or even reverse this process. In addition, as TN/TX inhibition may also elevate other microRNAs, it might be more advantageous to explore other strategies to increase miR-181b levels selectively. For example, recent studies have demonstrated that methotrexate increases transcription of miR-181b since its metabolite, adenosine, stimulates the adenosine A3 receptor (Yang et al., 2021). Focusing on strategies able to selectively elevate miR-181b seems particularly attractive since, in addition to its ability to decrease stiffness in VSMCs, this microRNA also exerts anti-inflammatory effects in endothelial cells (Sun et al., 2014). However, since extremely high elevations of miR-181b have been implicated in the development of atherosclerotic plaques, (Di Gregoli et al., 2017), it may be important to identify strategies that reverse the age-associated decline in miR-181b levels without elevating them further into the range associated with pathological effects.

In summary, the hypothesis that microRNAs play a key role in the pathophysiology of LAS has stimulated important progress in identifying specific microRNAs implicated in this process. These advances provide a firm foundation for further studies aimed at deciphering: (1) how these microRNAs are regulated, and (2) how they impact arterial stiffness, information that will be essential to identifying strategies capable of combatting the enormous public health challenge presented by LAS.

## Author Contributions

JB, ET, DB, and SD discussed the scope of the review. JB wrote the initial draft and revised it based on comments made by ET. All authors contributed to the article and approved the submitted version.

## Funding

This study was supported by NIH, Stimulating and Advancing ACCM Research, Western Institute for Veterans Research.

## Conflict of Interest

The authors declare that the research was conducted in the absence of any commercial or financial relationships that could be construed as a potential conflict of interest.

## Publisher's Note

All claims expressed in this article are solely those of the authors and do not necessarily represent those of their affiliated organizations, or those of the publisher, the editors and the reviewers. Any product that may be evaluated in this article, or claim that may be made by its manufacturer, is not guaranteed or endorsed by the publisher.
